# Does Provider Identity at Triage Improve Machine Learning Prediction of Hospital Admission? A Comparative Analysis of Ten Supervised Classifiers with SHAP Explainability

**DOI:** 10.3390/jpm16040204

**Published:** 2026-04-05

**Authors:** Adam E. Brown, Chance W. Marostica, Wayne A. Martini

**Affiliations:** 1 Mayo Clinic Alix School of Medicine, Mayo Clinic, Scottsdale, AZ 85259, USA; 2 Department of Emergency Medicine, Mayo Clinic, 5777 E Mayo Blvd, Phoenix, AZ 85054, USA

**Keywords:** hospital admission prediction, emergency department, triage, machine learning, provider variation, explainable artificial intelligence, SHAP, CatBoost, XGBoost, personalized medicine

## Abstract

**Background/Objectives:** Machine learning (ML) models can predict hospital admission from emergency department (ED) triage data with areas under the receiver operating characteristic curve (AUC) exceeding 0.85. Whether incorporating the assigned provider’s identity—as a proxy for unmeasured practice variation—improves prediction has not been systematically studied. We aimed to compare 10 supervised ML classifiers for predicting hospital admission at ED triage, with and without provider identity, and to characterize model reasoning using SHapley Additive exPlanations (SHAP). **Methods:** We conducted a retrospective cohort study of 186,094 ED visits (2020–2023, training) and 58,151 visits (2024, temporal holdout test) at one academic tertiary-care ED. Ten classifiers spanning linear, distance-based, tree-based, ensemble, probabilistic, and neural network families were each trained in two conditions: baseline (23 triage features) and with provider identity appended. SHAP TreeExplainer was applied to the top-performing models (CatBoost and XGBoost). **Results:** The admission rate was 31.3% (training) and 31.7% (test). CatBoost achieved the highest baseline AUC of 0.8906 (0.8878–0.8933). Adding provider identity produced negligible AUC changes across all models (ΔAUC range: −0.0029 to +0.0015; all DeLong *p* > 0.05). SHAP analysis identified ESI level, respiratory rate, temperature, complaint category, and age as the dominant predictors, with clinically intuitive directionality. **Conclusions:** Provider identity does not meaningfully improve ML prediction of hospital admission beyond standard triage variables. The observed 28-percentage-point variation in provider admission rates is explained by patient case-mix differences than with independent practice pattern effects on prediction. SHAP provides transparent, clinically interpretable explanations suitable for bedside decision support.

## 1. Introduction

Emergency department (ED) crowding remains a persistent and worsening challenge in the United States and internationally, contributing to prolonged wait times, boarding of admitted patients in hallways, diversion of ambulances, and adverse patient outcomes [1,2]. An estimated 145 million ED visits occur annually in the United States, and the mismatch between demand for ED care and available inpatient beds is a central driver of crowding [1,3]. Beyond compromising patient safety, ED crowding has been associated with increased mortality, longer hospital stays, and higher rates of patients leaving without being seen [2,4]. Making early identification of patients who will require hospital admission a clinical and operational priority. Accurate early prediction of admission at the time of triage could facilitate proactive bed requests, earlier disposition planning, and more efficient allocation of ED resources, particularly nursing staff and monitored beds.

Traditional triage systems such as the Emergency Severity Index (ESI) assign an acuity level from 1 (highest) to 5 (lowest) based on initial presentation, predicted resource utilization, and the triage nurse’s clinical judgment [5]. While ESI effectively stratifies patients by illness severity, it is a coarse five-level tool designed primarily to prioritize care rather than to predict disposition. A substantial proportion of patients triaged at ESI level 3—the most common category—are ultimately admitted to the hospital, indicating that standard triage acuity alone does not reliably identify all patients requiring admission [6]. This limitation motivates the development of more nuanced, data-driven decision support tools that can augment clinical judgment at triage.

Machine learning (ML) models trained on structured triage data have demonstrated promising accuracy for predicting hospital admission, with reported areas under the receiver operating characteristic curve (AUC) in the 0.80–0.90 range [7,8,9,10,11]. These models typically use demographics, vital signs, triage acuity scores such as the ESI, mode of arrival, and chief complaint to generate predictions at or near the time of patient arrival. Parker et al. (2019) developed a novel prediction model using eight triage variables and reported an AUC of 0.83 [7]. Fernandes et al. (2020) applied ML including natural language processing of chief complaints to predict ED admissions, achieving an AUC of 0.91 [8]. Ryu et al. (2022) assessed the generalizability of a clinical ML model across multiple EDs and found AUCs ranging from 0.80 to 0.85 depending on the site [9]. Prior work has established that gradient-boosted tree models (e.g., XGBoost, CatBoost) generally outperform traditional approaches such as logistic regression, particularly when handling nonlinear interactions among clinical variables [8,10,11]. However, the comparative performance of a broad range of ML classifier families for admission prediction has not been comprehensively evaluated in a single study using the same dataset and evaluation methodology.

An underexplored factor in ED admission prediction is the role of the treating provider. In most EDs, a physician or advanced practice provider is assigned to a patient at or near the time of triage completion. Provider practice patterns are known to vary substantially with respect to admission thresholds, diagnostic test ordering, and disposition decisions [12,13,14]. Studies have documented significant inter-physician variation in admission rates for common ED presentations such as chest pain and pneumonia, even after adjusting for patient acuity [14]. Venkatesh et al. (2015) analyzed over 29 million ED visits and found that hospital-level admission rates varied from 14% to 72% across institutions, with substantial within-hospital provider-level variation as well [12]. Dean et al. (2012) found that individual physician admission rates for community-acquired pneumonia varied by a factor of two even within a single ED [14]. If provider identity captures unmeasured patient or system factors, such as differential assignment of sicker patients to more experienced physicians, systematic variation in clinical decision-making, or provider-specific admission thresholds, it could serve as a useful additional predictor of admission. Conversely, if structured triage data already capture the clinical severity that drives admission decisions, provider identity may be redundant and offer no incremental predictive value.

Furthermore, a critical barrier to clinical adoption of ML models in emergency medicine is the lack of interpretability. Clinicians are unlikely to trust or act upon predictions they cannot understand [15,16]. SHapley Additive exPlanations (SHAP) [17] provide a theoretically grounded framework for decomposing predictions into feature-level contributions, offering population-level importance rankings that align with clinical reasoning. Unlike permutation importance or simple feature coefficients, SHAP values satisfy several desirable properties including local accuracy, missingness, and consistency [17]. SHAP has been increasingly applied in clinical prediction models to provide transparent explanations that can be evaluated by domain experts [18,19]. From a personalized medicine perspective, SHAP-based explanations can highlight which features are most influential for a given patient’s prediction, enabling clinicians to evaluate whether the model’s reasoning aligns with their clinical assessment. This transparency is critical for responsible integration of ML into emergency care workflows and for building the clinician trust necessary for adoption [15,16].

In this study, we compared 10 supervised ML classifiers—spanning linear models, distance-based methods, tree-based ensembles, and a neural network—for predicting hospital admission at a single academic ED. Each model was trained in two conditions: once using only standard triage features and once with the assigned provider included. We additionally performed SHAP explainability analysis on the top-performing models to characterize population-level feature contributions. We hypothesized that (1) gradient-boosted tree models would outperform other classifier families, (2) provider identity would not meaningfully improve prediction beyond standard triage variables, and (3) SHAP analysis would identify clinically intuitive features as the dominant predictors.

## 2. Materials and Methods

### 2.1. Study Design and Setting

We performed a retrospective cohort study of ED visits from 1 January 2020, through 31 December 2024, at a single academic tertiary-care ED (Mayo Clinic, Phoenix, AZ, USA). This high-volume ED treats approximately 55,000 patients per year and uses the Epic electronic health record (EHR) system (Epic Systems V100.2602.1.0, Verona, WI, USA). Triage is performed by registered nurses using the ESI five-level triage system. The study included all patients evaluated in the ED who were subsequently admitted to the hospital (including observation status) or discharged home. Encounters resulting in other dispositions (left without being seen, transfer to an outside facility, or dead on arrival) were excluded. The study was approved by the Mayo Clinic Institutional Review Board (IRB #25-002035, approved 22 February 2026) with a waiver of informed consent given the minimal-risk, retrospective design.

### 2.2. Cohort Construction and Temporal Split

All data were extracted from the EHR data warehouse via structured query language (SQL) queries. The training cohort consisted of all eligible ED visits from 1 January 2020, through 31 December 2023 (four calendar years). A temporally distinct holdout test set was constructed from all visits between 1 January 2024, and 31 December 2024. This temporal split ensures that model performance is evaluated on entirely novel data from a different calendar year, guarding against temporal data leakage and providing a realistic estimate of prospective performance. The binary outcome was hospital admission (inpatient admission or observation status) versus ED discharge. The final analytic dataset comprised 186,094 training encounters and 58,151 test encounters.

### 2.3. Baseline Features

All predictor variables were structured data available at the time of triage completion, prior to any diagnostic testing or physician evaluation. Baseline features included: patient age (continuous), sex (binary), Emergency Severity Index (ESI) triage level (ordinal, 1–5), complaint category (categorical), mode of arrival (binary flags for ambulance and wheel-chair arrival), six triage vital signs (systolic blood pressure [SBP], diastolic blood pressure [DBP], heart rate, respiratory rate, peripheral oxygen saturation [SpO_2_], and temperature), and nine binary comorbidity flags derived from the EHR problem list: heart failure, myocardial infarction or coronary artery disease, diabetes mellitus, chronic kidney disease, COPD or asthma, active cancer or malignancy, transient ischemic attack or stroke, pulmonary embolism, and organ transplant history. In total, the baseline feature set comprised 23 variables.

Comorbidities were derived from each patient’s active problem list and prior diagnosis codes in the EHR using keyword and ICD-10 code matching. Each comorbidity was represented as a binary variable (present/absent). Complaint category was a structured field recorded by the triage nurse from a predefined list of 20 categories in the EHR; the five most common categories were musculoskeletal/trauma (19.6%), abdominal/GI (18.8%), other/unspecified (17.4%), respiratory (9.6%), and cardiac/chest pain (9.2%). ESI levels were assigned by triage nurses according to the standard ESI algorithm [5].

### 2.4. Missing Data

Missing values for vital signs were minimal. In the training cohort, the missing data rates were systolic blood pressure 0.37%, diastolic blood pressure 0.37%, heart rate 0.80%, respiratory rate 0.37%, oxygen saturation 0.08%, and temperature 1.07%. ESI level was missing in 0.03% of encounters. No encounters had missing age or complaint category data. Missing vital signs were imputed using training-set medians for models that do not handle missing data natively; CatBoost handled missing values internally. Categorical variables (ESI level, complaint category) were label-encoded for most models; CatBoost handled them as native categoricals.

### 2.5. Provider Feature

The provider feature was defined as the first ED provider assigned to the patient, as recorded in the EHR. At this institution, a provider is typically assigned at or near the time of arrival. For models other than CatBoost, provider identity was encoded using target encoding: each provider was represented by their mean admission rate calculated exclusively from the training data. Providers appearing in the test set but not in the training set were assigned the global mean admission rate. CatBoost handled the provider as a native categorical feature. In total, there were 66 unique providers in the training set and 49 in the test set.

### 2.6. Models

We evaluated 10 supervised classifiers spanning the major families of ML algorithms: Logistic Regression (L2-regularized, SAGA solver), K-Nearest Neighbors (KNN, k = 15), linear-kernel Support Vector Machine (SVM) with Platt calibration, Decision Tree (max depth 10), Random Forest (200 trees, max depth 15), XGBoost (300 trees, depth 6, learning rate 0.1), CatBoost (500 iterations, depth 6, learning rate 0.1), AdaBoost (200 estimators), Gaussian Naïve Bayes, and Multi-Layer Perceptron (MLP; two hidden layers of 128 and 64 units with early stopping). Models requiring scaled numeric input (Logistic Regression, KNN, SVM, Naïve Bayes, MLP) were trained on standardized features. Each model was trained in two conditions: baseline (23 features) and with provider (24 features), yielding 20 model–condition combinations.

We selected these 10 classifiers to represent linear models (Logistic Regression, SVM), distance-based methods (KNN), tree-based methods (Decision Tree), ensemble methods (Random Forest, XGBoost, CatBoost, AdaBoost), probabilistic models (Naïve Bayes), and neural networks (MLP). Hyperparameters for each model were selected based on commonly used defaults and prior literature recommendations for clinical tabular data [8,10,11]. Hyperparameters were not optimized via grid search or Bayesian optimization; the focus of this study was on comparative classifier performance and explainability rather than maximizing any single model’s performance. A complete table of hyperparameters is provided in Table S3 (Supplementary Materials).

### 2.7. Evaluation Metrics and Threshold Optimization

The primary discriminative metric was AUC. Secondary metrics included precision–recall AUC (PR AUC), accuracy, sensitivity (recall), specificity, positive predictive value (PPV), negative predictive value (NPV), F1 score, and Brier score. For each model, the classification threshold was optimized on the training data by selecting the value in the range 0.20–0.80 that maximized the F1 score. This threshold was applied to the holdout test set without further adjustment.

Bootstrap 95% confidence intervals (CIs) for AUC were computed using 2000 bootstrap resamples of the test set. To assess whether differences in AUC between models were statistically significant, we performed approximate DeLong tests [20] comparing each model’s baseline versus provider-augmented AUC and comparing CatBoost to each other baseline model. A two-sided *p*-value < 0.05 was considered statistically significant. All analyses were conducted using Python 3.13 with scikit-learn, XGBoost, and CatBoost.

### 2.8. Explainability Analysis

To assess feature contributions and model interpretability, we applied SHAP [17] to the two top-performing baseline models (CatBoost and XGBoost). We used TreeExplainer, which computes exact SHAP values for tree-based models in polynomial time, unlike KernelExplainer which requires approximation [17]. SHAP values were computed on a random subsample of 3000 test-set encounters, selected to balance computational efficiency with representative coverage. Population-level explainability was assessed via SHAP summary (beeswarm) plots, which display the distribution of each feature’s impact colored by feature value, and mean absolute SHAP bar plots quantifying overall feature importance.

## 3. Results

### 3.1. Cohort Characteristics

The training cohort included 186,094 ED visits, of which 58,155 (31.3%) resulted in hospital admission and 127,939 (68.7%) were discharged. The temporally distinct test cohort (2024) included 58,151 visits with 18,443 (31.7%) admissions, confirming a stable admission rate across time periods. Table 1 presents the complete demographic comparison.

Admitted patients were significantly older (training: median 68 years, IQR 54–78 vs. 57 years, IQR 37–71), less likely to be female (47.4% vs. 56.0%), and substantially more likely to arrive by ambulance (15.9% vs. 4.9%). 49.5% of admitted patients had ESI 1–2 compared with 17.0% of discharged patients. All comparisons between admitted and discharged patients were statistically significant (*p* < 0.001 for all variables by Mann–Whitney U test for continuous variables and chi-square test for categorical variables), reflecting both the large sample size and clinically meaningful differences between groups.

### 3.2. Provider Variation

**Figure 1:**
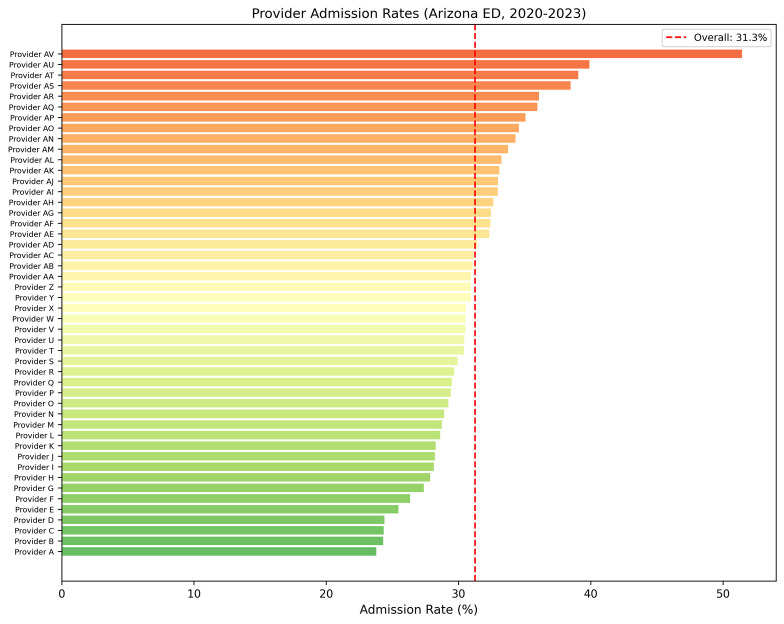
Hospital admission rates by individual emergency department provider, Arizona ED training cohort (2020–2023). Providers are de-identified and ranked in ascending order of admission rate; only clinicians with ≥100 triage encounters are shown (n = 48). Each bar represents the proportion of a provider’s patients admitted to the hospital, with color gradient from green (lowest) to red (highest). The dashed red line denotes the overall cohort admission rate of 31.3%. Observed provider-level rates ranged from approximately 24% to 52%, a roughly two-fold spread that motivated evaluation of provider identity as a candidate feature in the supervised classifiers assessed in this study.

Among the 66 providers in the training cohort, hospital admission rates ranged from 23.8% to 51.4% for all providers with ≥100 encounters. For the 44 high-volume providers (those with ≥1000 encounters), rates ranged from 24.3% to 39.9%, with a mean of 31.2% (SD 3.5%), representing a 15.6-percentage-point spread. The distribution was approximately normal, with most providers clustering near the overall rate of 31.3% (Figure 1).

### 3.3. Model Performance: Baseline

**Figure 2:**
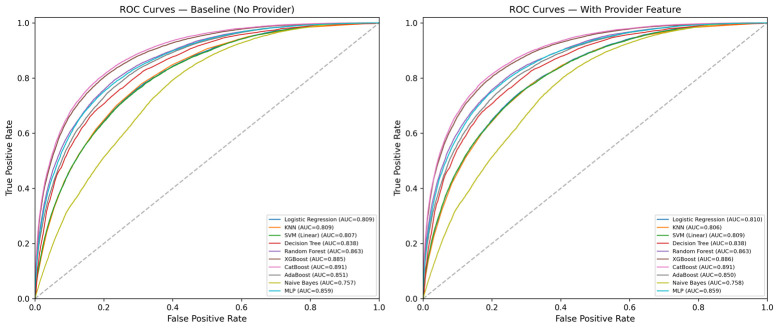
AUC Comparison Across Ten Classifiers With and Without Provider Identity as a Feature. Receiver operating characteristic (ROC) curves for all ten supervised classifiers evaluated on the holdout test set. Left panel: baseline models trained without the provider identity feature. Right panel: identical models retrained with provider identity included as an input feature. Area under the curve (AUC) values are reported in each legend. CatBoost (AUC = 0.891) and XGBoost (AUC = 0.885 baseline/0.886 with provider) achieved the highest discrimination in both conditions, while Naïve Bayes performed worst (AUC ≈ 0.757–0.758), consistent with violation of its feature-independence assumption. The diagonal dashed gray line denotes chance-level performance. Across every classifier, inclusion of the provider feature produced a change in AUC of ≤0.003, demonstrating that provider identity at triage contributes negligible additional discriminative information beyond the routinely captured clinical and demographic variables.

Without the provider feature, CatBoost achieved the highest AUC of 0.8906 (0.8878–0.8933) on the holdout test set, with sensitivity of 76.5% and specificity of 84.3% at the optimized threshold (Table 2). XGBoost was second (0.8845 (0.8816–0.8873)), followed by Random Forest (0.8633 (0.8600–0.8663)), MLP (0.8607 (0.8574–0.8637)), and AdaBoost (0.8509 (0.8476–0.8540)). Among linear models, Logistic Regression (0.8086 (0.8048–0.8123)) and SVM (0.8074 (0.8035–0.8111)) achieved similar performance. Naïve Bayes had the weakest discrimination (0.7576 (0.7536–0.7617)). DeLong tests confirmed that CatBoost significantly outperformed all other baseline models (all *p* < 0.001), including XGBoost (z = 4.111, *p* < 0.001). Figure 2 shows the ROC curves.

### 3.4. Effect of Adding Provider Identity

**Figure 3:**
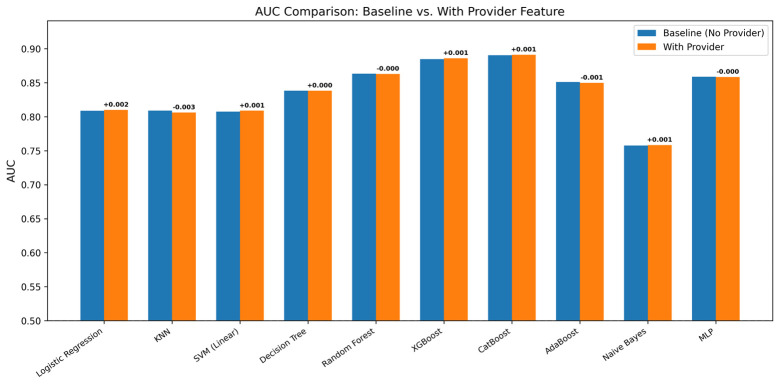
AUC Comparison Across Ten Classifiers With and Without Provider Identity as a Feature: Side-by-side comparison of area under the receiver operating characteristic curve (AUC) for all ten classifiers, with (orange) and without (blue) provider identity as an input feature. Numeric delta values (ΔAUC = with-provider − baseline) are annotated above each bar pair. The largest observed change across any model was |ΔAUC| = 0.003 (KNN), with eight of ten classifiers showing changes of ≤0.001. No model demonstrated a clinically or statistically meaningful improvement in discrimination when provider identity was added, supporting the conclusion that treating-provider identity at triage does not encode predictive signal beyond what is already captured by standard clinical and demographic features.

Adding the provider feature produced minimal changes in AUC across all 10 classifiers (Table 3). The largest positive ΔAUC was +0.0015 and the largest negative ΔAUC was −0.0029. All ΔAUC values fell within ±0.003. For CatBoost, AUC changed from 0.8906 to 0.8911 (ΔAUC = +0.0005). CatBoost—which handled the provider as a native categorical variable rather than target encoding—showed a similarly negligible effect, ruling out encoding artifacts. Figure 3 shows the AUC comparison. 

Approximate DeLong tests confirmed that none of the baseline-versus-provider AUC differences were statistically significant (Logistic Regression *p* = 0.42; KNN *p* = 0.12; SVM *p* = 0.41; Decision Tree *p* = 0.96; Random Forest *p* = 0.87; XGBoost *p* = 0.45; CatBoost *p* = 0.75; AdaBoost *p* = 0.52; Naïve Bayes *p* = 0.67; MLP *p* = 0.26), providing formal statistical support for the null finding. The target-encoded provider feature alone achieved a standalone AUC of only 0.5346, barely above random chance.

### 3.5. Feature Importance and Explainability

SHAP analysis of the CatBoost baseline model revealed the following top five features by mean absolute SHAP value: Respiratory Rate, ESI Level, Temperature, Complaint Category, Age. For XGBoost, the top five features were: ESI Level, Respiratory Rate, Temperature, Complaint Category, Age. The two models showed highly concordant feature importance hierarchies (Table 4). Vital signs collectively dominated, consistent with clinical expectations that acuity is the primary driver of admission decisions.

**Figure 4:**
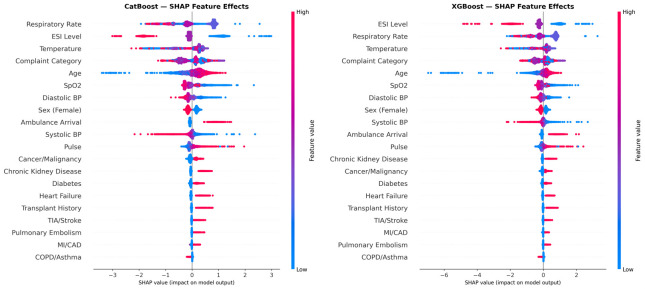
SHAP beeswarm plots for the two top-performing baseline models (no provider feature) on a random sample of 3000 holdout test-set encounters.

The SHAP beeswarm plots (Figure 4) reveal directionality: higher ESI acuity (lower ESI number), elevated respiratory rate, abnormal temperature, and lower oxygen saturation pushed predictions toward admission. Older age increased admission risk; female sex had a modest protective association. Among comorbidities, cancer/malignancy and chronic kidney disease had the highest SHAP importance. Notably, 48.9% of admitted patients in the training cohort had an ESI of 3, compared with 64.7% of discharged patients—this overlap underscores why ESI alone is insufficient and why ML models integrating multiple features provide superior discrimination.

## 4. Discussion

In this single-site retrospective study comparing 10 supervised ML classifiers, we found that adding provider identity as a feature at ED triage did not meaningfully improve prediction of hospital admission. Despite substantial observable variation in admission rates across providers (24.3% to 39.9% for high-volume providers; mean 31.2%, SD 3.5%), this variation did not translate into incremental predictive value when combined with standard triage data. The finding was remarkably consistent across all model families—from linear models and naïve Bayes to gradient-boosted ensembles and a neural network—with no model showing a ΔAUC exceeding ±0.003. Formal DeLong tests confirmed that none of these differences were statistically significant (all *p* > 0.05).

The null result for the provider feature has several explanations. First, provider-level admission rate differences likely reflect case-mix variation rather than true practice pattern variation orthogonal to measured features. Diagnostic analysis confirmed that the target-encoded provider feature has a standalone AUC of only 0.535—barely above random—and a residual correlation of 0.059 with baseline model errors, indicating negligible signal beyond what the model already captures [18]. Second, the assignment of providers to patients at triage may be largely random with respect to unmeasured acuity; at this institution, provider assignment is typically based on pod staffing and patient flow rather than clinical severity. Third, even if genuine practice variation exists, it may operate on the margin of disposition decisions (e.g., ESI 3 patients with equivocal indications) where the signal is too weak to be captured by aggregate target encoding.

Consistent with prior literature [8,10,11], gradient-boosted models outperformed all other classifiers. CatBoost achieved the highest AUC (0.8906, 95% CI: 0.8878–0.8933), likely benefiting from native categorical handling and ordered boosting [21]. This performance is comparable to or exceeds published benchmarks: Parker et al. (2019) reported an AUC of 0.83 using 8 variables [7], Raita et al. (2019) achieved 0.82 on NHAMCS data [22], Ryu et al. (2022) reported 0.80–0.85 across multiple sites [9], and Fenn et al. (2021) achieved 0.85 with gradient-boosted trees [23]. Our higher AUC may reflect the benefit of a larger training cohort (186,094 encounters), the inclusion of 9 comorbidity features, and CatBoost’s native handling of categorical variables. The near-identical performance of Logistic Regression, SVM, and KNN suggests that the linear approximation captures most signal in triage data, with gradient-boosted models extracting additional value from nonlinear interactions.

The SHAP analysis provides population-level insight into model reasoning. The dominance of ESI level, respiratory rate, temperature, and complaint category aligns with known clinical drivers of admission, lending face validity. The concordance between CatBoost and XGBoost feature importance rankings suggests these are robust, model-agnostic patterns rather than artifacts of a specific algorithm [17,18].

From a personalized medicine perspective, SHAP-based feature importance provides a framework for transparent clinical decision support. Rather than presenting a single probability, the model can highlight which features are most influential for a given patient population—enabling clinicians to evaluate whether the model’s reasoning aligns with clinical expectations. For example, in a patient presenting with an ESI level 3, elevated respiratory rate, and active malignancy, SHAP analysis can show that the respiratory rate and cancer history are the primary drivers pushing the prediction toward admission, providing an interpretable rationale that mirrors clinical reasoning. This transparency is critical for responsible integration of ML into emergency care workflows. In the context of precision emergency medicine, such models could be embedded into electronic health record-based clinical decision support tools that surface patient-specific risk profiles at triage, supporting individualized disposition planning and resource allocation [15,16,24].

This study has several limitations. First, it was conducted at a single academic ED; provider practice patterns may vary more at community hospitals or safety-net institutions, potentially making the provider feature more informative in those settings. Second, we used target encoding for the provider feature; alternative approaches such as entity embeddings or mixed-effects models might capture more nuanced provider effects, though the consistently null result across CatBoost’s native categorical handling makes this unlikely to change the conclusions. Third, we did not account for temporal changes in provider panels or within-provider variation; patients may be transferred between providers during the visit, and only the first assigned provider was captured. Fourth, hyperparameters were not extensively tuned via grid search or Bayesian optimization, which may have slightly underestimated the performance of some models; however, because the same approach was applied to all classifiers, relative comparisons remain valid. Fifth, the study population was drawn from a single academic medical center, limiting generalizability to more diverse settings. Additionally, the training cohort (2020–2023) spans the COVID-19 pandemic period; however, the stable admission rate between training (31.3%) and test (31.7%) cohorts and the consistency of demographic characteristics across time periods (Table 1) suggest that pandemic-related shifts did not meaningfully affect model generalizability. Sixth, we did not compare model performance against existing clinical prediction rules or validated scoring systems for hospital admission. Finally, provider identity might carry more value for specific disposition subtypes or other outcomes.

Future work should evaluate the provider effect in multicenter datasets with greater provider diversity and explore provider-level features beyond raw identity (years of experience, specialty training, historical patterns for specific complaint categories). Prospective validation of the admission prediction model in a real-time clinical workflow is a critical next step, as retrospective performance may not fully translate to the clinical environment. Integration of natural language processing of free-text triage notes and chief complaints could further improve model performance. Subgroup analyses by ESI level and complaint category could reveal clinical scenarios where ML-augmented prediction provides the greatest value over standard triage. Fairness auditing across demographic groups (age, sex, race/ethnicity) should be performed before any clinical deployment to ensure equitable model performance. Finally, the SHAP explainability framework demonstrated here could be extended with individual patient-level waterfall plots for real-time bedside decision support [24,25].

## 5. Conclusions

In a comparative analysis of 10 supervised ML classifiers at a single academic ED, provider identity did not meaningfully improve prediction of hospital admission beyond standard triage variables (maximum |ΔAUC| = 0.0029; all DeLong test *p* > 0.05). CatBoost achieved the highest overall performance (AUC 0.8906, 95% CI: 0.8878–0.8933). SHAP analysis confirmed that ESI level, respiratory rate, and temperature are the dominant predictors of admission, with clinically intuitive feature importance rankings concordant across both top-performing models. Admission prediction models can be confidently developed using triage data alone, without incorporating provider identity. The SHAP-based explainability framework provides a path toward transparent, trustworthy ML deployment in emergency medicine and supports the broader goal of precision emergency medicine through individualized, interpretable clinical decision support.

## Figures and Tables

**Table 1:** Baseline characteristics of training (2020–2023) and test (2024) cohorts, stratified by disposition. [See original Table 1—add *p*-value column showing *p* < 0.001 for all rows.].

Characteristic	Train Admitted	Train Discharged	Test Admitted	Test Discharged	*p*-Value *
	(*n* = 58,155)	(*n* = 127,939)	(*n* = 18,443)	(*n* = 39,708)	
Age, median (IQR)	68 (54–78)	57 (37–71)	68 (54–78)	58 (37–73)	<0.001 †
Female, n (%)	27,560 (47.4%)	71,645 (56.0%)	8947 (48.5%)	22,538 (56.8%)	<0.001 ‡
Ambulance arrival, *n* (%)	9259 (15.9%)	6221 (4.9%)	2888 (15.7%)	1902 (4.8%)	<0.001 ‡
ESI 1, n (%)	1135 (2.0%)	153 (0.1%)	245 (1.3%)	28 (0.1%)	<0.001 ‡
ESI 2, n (%)	27,625 (47.5%)	21,624 (16.9%)	8143 (44.2%)	5839 (14.7%)	
ESI 3, n (%)	28,434 (48.9%)	82,771 (64.7%)	9688 (52.5%)	25,421 (64.0%)	
ESI 4, n (%)	939 (1.6%)	22,289 (17.4%)	358 (1.9%)	8143 (20.5%)	
ESI 5, n (%)	3 (0.0%)	1063 (0.8%)	8 (0.0%)	262 (0.7%)	
SBP, mmHg, median (IQR)	126 (114–140)	129 (117–143)	126 (114–140)	130 (117–144)	<0.001 †
DBP, mmHg, median (IQR)	74 (65–83)	78 (70–87)	74 (65–83)	79 (70–87)	<0.001 †
Heart rate, bpm, median (IQR)	76 (67–87)	75 (66–86)	76 (67–86)	74 (66–85)	<0.001 †
Respiratory rate, median (IQR)	16 (16–18)	17 (16–18)	16 (16–18)	18 (16–18)	<0.001 †
SpO_2_, %, median (IQR)	97 (95–98)	98 (96–99)	97 (95–98)	97 (96–98)	<0.001 †
Temperature, °F, median (IQR)	98.1 (97.7–98.4)	98.1 (97.7–98.4)	98.1 (97.7–98.2)	98.0 (97.6–98.4)	<0.001 †
Heart failure, n (%)	9726 (16.7%)	8048 (6.3%)	2688 (14.6%)	2196 (5.5%)	<0.001 ‡
MI/CAD, n (%)	10,839 (18.6%)	12,351 (9.7%)	3312 (18.0%)	3607 (9.1%)	<0.001 ‡
Diabetes, n (%)	16,626 (28.6%)	21,774 (17.0%)	5126 (27.8%)	6723 (16.9%)	<0.001 ‡
CKD, n (%)	10,822 (18.6%)	9652 (7.5%)	2985 (16.2%)	2731 (6.9%)	<0.001 ‡
COPD/Asthma, n (%)	8306 (14.3%)	19,650 (15.4%)	2814 (15.3%)	6079 (15.3%)	<0.001 ‡
Cancer/Malignancy, n (%)	23,276 (40.0%)	33,498 (26.2%)	7512 (40.7%)	10,460 (26.3%)	<0.001 ‡
TIA/Stroke, n (%)	8652 (14.9%)	10,140 (7.9%)	2441 (13.2%)	2958 (7.4%)	<0.001 ‡
PE, n (%)	4533 (7.8%)	6047 (4.7%)	1546 (8.4%)	1979 (5.0%)	<0.001 ‡
Transplant history, n (%)	4296 (7.4%)	3265 (2.6%)	1120 (6.1%)	868 (2.2%)	<0.001 ‡

* *p*-values compare admitted vs. discharged in the training cohort. † Mann–Whitney U test. ‡ Chi-square test. IQR = interquartile range; ESI = Emergency Severity Index; SBP = systolic blood pressure; DBP = diastolic blood pressure; SpO_2_ = oxygen saturation; CKD = chronic kidney disease; COPD = chronic obstructive pulmonary disease; TIA = transient ischemic attack; PE = pulmonary embolism.

**Table 2:** Performance of 10 classifiers on the holdout test set (baseline). AUC = area under the receiver operating characteristic curve with 95% bootstrap CI; PR AUC = precision–recall AUC; Acc. = accuracy; Sens. = sensitivity; Spec. = specificity; PPV = positive predictive value; NPV = negative predictive value; Brier = Brier score (lower is better).

Model	AUC (95% CI)	PR AUC	Acc.	Sens.	Spec.	PPV	NPV	F1	Brier
Logistic Regression	0.8086 (0.8048–0.8123)	0.6557	0.7269	0.7471	0.7176	0.5513	0.8593	0.6344	0.1614
KNN	0.8090 (0.8053–0.8126)	0.6349	0.7187	0.7790	0.6907	0.5391	0.8706	0.6372	0.1607
SVM (Linear)	0.8074 (0.8035–0.8111)	0.6560	0.7212	0.7537	0.7061	0.5436	0.8606	0.6316	0.1618
Decision Tree	0.8381 (0.8345–0.8414)	0.6953	0.7629	0.7348	0.7760	0.6037	0.8630	0.6628	0.1480
Random Forest	0.8633 (0.8600–0.8663)	0.7564	0.7831	0.7696	0.7894	0.6293	0.8806	0.6924	0.1379
XGBoost	0.8845 (0.8816–0.8873)	0.7916	0.8110	0.7678	0.8311	0.6786	0.8851	0.7204	0.1254
CatBoost	0.8906 (0.8878–0.8933)	0.8009	0.8181	0.7654	0.8426	0.6931	0.8855	0.7275	0.1228
AdaBoost	0.8509 (0.8476–0.8540)	0.7295	0.7736	0.7512	0.7840	0.6176	0.8715	0.6779	0.2058
Naïve Bayes	0.7576 (0.7536–0.7617)	0.5474	0.7035	0.5507	0.7745	0.5314	0.7877	0.5409	0.2502
MLP	0.8607 (0.8574–0.8637)	0.7462	0.7856	0.7467	0.8036	0.6385	0.8723	0.6884	0.1382

**Table 3:** AUC comparison: baseline versus with provider feature. ΔAUC = change in AUC when provider is added. DeLong *p*-value tests whether the change is statistically significant.

Model	Baseline AUC (95% CI)	With Provider AUC (95% CI)	ΔAUC	DeLong *p*
Logistic Regression	0.8086 (0.8048–0.8123)	0.8101 (0.8063–0.8137)	+0.0015	0.42
KNN	0.8090 (0.8053–0.8126)	0.8061 (0.8023–0.8098)	−0.0029	0.12
SVM (Linear)	0.8074 (0.8035–0.8111)	0.8089 (0.8051–0.8126)	+0.0015	0.41
Decision Tree	0.8381 (0.8345–0.8414)	0.8382 (0.8346–0.8415)	+0.0001	0.96
Random Forest	0.8633 (0.8600–0.8663)	0.8630 (0.8597–0.8660)	−0.0003	0.87
XGBoost	0.8845 (0.8816–0.8873)	0.8856 (0.8827–0.8884)	+0.0012	0.45
CatBoost	0.8906 (0.8878–0.8933)	0.8911 (0.8883–0.8938)	+0.0005	0.75
AdaBoost	0.8509 (0.8476–0.8540)	0.8498 (0.8465–0.8528)	−0.0011	0.52
Naïve Bayes	0.7576 (0.7536–0.7617)	0.7585 (0.7545–0.7626)	+0.0009	0.67
MLP	0.8607 (0.8574–0.8637)	0.8588 (0.8557–0.8619)	−0.0002	0.26

**Table 4:** Top 10 features ranked by mean absolute SHAP value for the CatBoost and XGBoost baseline models (no provider feature). SHAP values were computed using TreeExplainer on a random sample of 3000 Arizona ED test-set encounters (seed = 42). Higher mean |SHAP| indicates greater average contribution to the model’s predicted probability of admission.

Rank	CatBoost Feature	Mean |SHAP|	XGBoost Feature	Mean |SHAP|
1	Respiratory Rate	0.6453	ESI Level	0.6848
2	ESI Level	0.6058	Respiratory Rate	0.6331
3	Temperature	0.4335	Temperature	0.4020
4	Complaint Category	0.3664	Complaint Category	0.3655
5	Age	0.3545	Age	0.3286
6	SpO2	0.2596	SpO2	0.2553
7	Diastolic BP	0.1871	Diastolic BP	0.1807
8	Sex (Female)	0.1676	Sex (Female)	0.1564
9	Ambulance Arrival	0.1476	Systolic BP	0.1545
10	Systolic BP	0.1465	Ambulance Arrival	0.1477

## Data Availability

The data presented in this study are not publicly available due to patient privacy restrictions under the institutional review board protocol. Requests for access to de-identified aggregate data may be directed to the corresponding author.

## Supplementary Materials

The following supporting information can be downloaded at https://www.mdpi.com/article/10.3390/jpm16040204/s1: Table S1: Full model performance metrics for all 20 model–condition combinations. Table S2: Complaint category frequencies. Table S3: Complete hyperparameter table for all 10 models. Table S4: Model specifications and hyperparameters for all 10 classifiers. Hyperparameters were set based on established defaults with empirical tuning on the training data. Models marked "Yes" for scaling were trained on features standardized to zero mean and unit variance using sklearn StandardScaler fitted exclusively on the training set. All random seeds were fixed at 42 for reproducibility. Table S5: Complete feature definitions for all predictor variables. The first 21 features (Age through Transplant History) plus Complaint Category constitute the 23-variable baseline feature set. The provider feature was appended as either a target-encoded continuous variable (for scikit-learn models and XGBoost) or a native categorical (for CatBoost). EHR = electronic health record; EMS = emergency medical services.Table S6: Overall cohort characteristics (not stratified by disposition) for training and test sets. The training cohort spans January 2020–December 2023; the test cohort spans January–December 2024. Admission rates were stable across time periods. IQR = interquartile range.
