# Supplementary material for: Does Provider Identity at Triage Improve Machine Learning Prediction of Hospital Admission? A Comparative Analysis of Ten Supervised Classifiers with SHAP Explainability
Source: J Pers Med. 2026 Apr 5;16(4):204. doi: 10.3390/jpm16040204 (PMC13117648; doi:10.3390/jpm16040204)
Supplement: Supplementary file 1 [file jpm-16-00204-s001.zip › Supplementary_Materials_JPM.pdf]

Supplementary Materials

Does Provider Identity at Triage Improve Machine Learning Prediction of Hospital Admission? A Comparative Analysis of Ten Supervised Classifiers with SHAP Explainability

Adam E. Brown, Chance M. Marostica and Wayne A. Martini

Journal of Personalized Medicine

Table S1. Full performance metrics for all 20 model–condition combinations on the temporal holdout test set (2024). Each of the 10 classifiers was trained in two conditions: Baseline (23 triage features only) and With Provider (24 features including target-encoded provider identity). The classification threshold was optimized on the training data to maximize the F1 score. AUC = area under the receiver operating characteristic curve; PR AUC = area under the precision–recall curve; PPV = positive predictive value; NPV = negative predictive value; Brier = Brier score (lower indicates better calibration).

Model	Condition	AUC	PR AUC	Accuracy	Sensitivity	Specificity	PPV	NPV	F1	Brier	Threshold
Logistic Regression	Baseline	0.8086	0.6557	0.7269	0.7471	0.7176	0.5513	0.8593	0.6344	0.1614	0.2800
Logistic Regression	With Provider	0.8101	0.6596	0.7268	0.7500	0.7160	0.5509	0.8605	0.6352	0.1605	0.2800
KNN	Baseline	0.8090	0.6349	0.7187	0.7790	0.6907	0.5391	0.8706	0.6372	0.1607	0.2700
KNN	With Provider	0.8061	0.6326	0.7156	0.7756	0.6878	0.5357	0.8684	0.6337	0.1616	0.2700
SVM (Linear)	Baseline	0.8074	0.6560	0.7212	0.7537	0.7061	0.5436	0.8606	0.6316	0.1618	0.2700
SVM (Linear)	With Provider	0.8089	0.6599	0.7272	0.7436	0.7197	0.5520	0.8580	0.6336	0.1609	0.2800
Decision Tree	Baseline	0.8381	0.6953	0.7629	0.7348	0.7760	0.6037	0.8630	0.6628	0.1480	0.3000
Decision Tree	With Provider	0.8382	0.6941	0.7627	0.7356	0.7753	0.6033	0.8632	0.6629	0.1481	0.3000
Random Forest	Baseline	0.8633	0.7564	0.7831	0.7696	0.7894	0.6293	0.8806	0.6924	0.1379	0.3200
Random Forest	With Provider	0.8630	0.7570	0.7852	0.7609	0.7965	0.6346	0.8776	0.6920	0.1380	0.3300
XGBoost	Baseline	0.8848	0.7916	0.8110	0.7678	0.8311	0.6786	0.8851	0.7204	0.1254	0.3200
XGBoost	With Provider	0.8860	0.7947	0.8083	0.7830	0.8201	0.6691	0.8905	0.7215	0.1245	0.3100
CatBoost	Baseline	0.8906	0.8009	0.8181	0.7654	0.8426	0.6931	0.8855	0.7275	0.1228	0.3300
CatBoost	With Provider	0.8911	0.8031	0.8180	0.7725	0.8391	0.6904	0.8881	0.7291	0.1221	0.3300
AdaBoost	Baseline	0.8509	0.7295	0.7736	0.7512	0.7840	0.6176	0.8715	0.6779	0.2058	0.4700

AdaBoost	With Provider	0.8498	0.7286	0.7708	0.7499	0.7804	0.6133	0.8705	0.6748	0.2055	0.4700
Naive Bayes	Baseline	0.7575	0.5474	0.7035	0.5507	0.7745	0.5314	0.7877	0.5409	0.2502	0.2000
Naive Bayes	With Provider	0.7584	0.5482	0.7042	0.5516	0.7751	0.5325	0.7882	0.5419	0.2498	0.2000
MLP	Baseline	0.8587	0.7462	0.7856	0.7467	0.8036	0.6385	0.8723	0.6884	0.1382	0.3300
MLP	With Provider	0.8585	0.7467	0.7859	0.7475	0.8038	0.6389	0.8727	0.6890	0.1393	0.3000

Table S2. Change in each performance metric when the provider feature is added (Δ = with provider minus baseline). Positive values indicate improvement. No model showed a clinically meaningful change in any metric. The largest absolute ΔAUC was 0.0029 (KNN, negative direction). Secondary metrics (PR AUC, accuracy, sensitivity, specificity, PPV, NPV, F1, Brier) confirmed the null result across all model families.

Model	ΔAUC	ΔPR AUC	ΔAccuracy	ΔSensitivity	ΔSpecificity	ΔPPV	ΔNPV	ΔF1	ΔBrier
Logistic Regression	+0.0015	+0.0039	-0.0001	+0.0029	-0.0016	-0.0004	+0.0012	+0.0008	-0.0009
KNN	-0.0029	-0.0023	-0.0031	-0.0034	-0.0029	-0.0034	-0.0022	-0.0035	+0.0009
SVM (Linear)	+0.0015	+0.0039	+0.0060	-0.0101	+0.0136	+0.0084	-0.0026	+0.0020	-0.0009
Decision Tree	+0.0001	-0.0012	-0.0002	+0.0008	-0.0007	-0.0004	+0.0002	+0.0001	+0.0001
Random Forest	-0.0003	+0.0006	+0.0021	-0.0087	+0.0071	+0.0053	-0.0030	-0.0004	+0.0001
XGBoost	+0.0012	+0.0031	-0.0027	+0.0152	-0.0110	-0.0095	+0.0054	+0.0011	-0.0009
CatBoost	+0.0005	+0.0022	-0.0001	+0.0071	-0.0035	-0.0027	+0.0026	+0.0016	-0.0007
AdaBoost	-0.0011	-0.0009	-0.0028	-0.0013	-0.0036	-0.0043	-0.0010	-0.0031	-0.0003
Naive Bayes	+0.0009	+0.0008	+0.0007	+0.0009	+0.0006	+0.0011	+0.0005	+0.0010	-0.0004
MLP	-0.0002	+0.0005	+0.0003	+0.0008	+0.0002	+0.0004	+0.0004	+0.0006	+0.0011

Table S3. Complete SHAP feature importance rankings for all features in the CatBoost and XGBoost baseline models. Mean |SHAP| values computed on a random subsample of 3000 test-set encounters using TreeExplainer. Features are ranked by descending mean absolute SHAP value. SHAP = SHapley Additive exPlanations. The top 10 features for both models are reported in the main manuscript (Table 4).

**Rank**	**CatBoost Feature**	**Mean |SHAP|**	**XGBoost Feature**	**Mean |SHAP|**
1	Respiratory Rate	0.650275	ESI Level	0.687067
2	ESI Level	0.599735	Respiratory Rate	0.633986
3	Temperature	0.511835	Temperature	0.404354
4	Complaint Category	0.410728	Complaint Category	0.371411
5	Age	0.358688	Age	0.332132
6	Oxygen Saturation	0.259323	Oxygen Saturation	0.254506
7	Diastolic BP	0.188667	Diastolic BP	0.182583
8	Sex (Female)	0.168569	Sex (Female)	0.157235
9	Heart Rate	0.163337	Systolic BP	0.151568
10	Ambulance Arrival	0.160318	Ambulance Arrival	0.146542
11	Systolic BP	0.145662	Heart Rate	0.142382
12	Cancer / Malignancy	0.084361	Chronic Kidney Disease	0.087283
13	Chronic Kidney Disease	0.084203	Cancer / Malignancy	0.081810
14	Diabetes	0.075969	Diabetes	0.077863
15	Heart Failure	0.069426	Heart Failure	0.067565
16	Transplant History	0.033019	Transplant History	0.032488
17	TIA / Stroke	0.030158	TIA / Stroke	0.031907
18	MI / CAD	0.021271	Pulmonary Embolism	0.021009
19	Pulmonary Embolism	0.021230	MI / CAD	0.020779
20	COPD / Asthma	0.018913	COPD / Asthma	0.020451
21	Wheelchair Arrival	0.009698	Wheelchair Arrival	0.010396

Table S4. Model specifications and hyperparameters for all 10 classifiers. Hyperparameters were set based on established defaults with empirical tuning on the training data. Models marked "Yes" for scaling were trained on features standardized to zero mean and unit variance using sklearn StandardScaler fitted exclusively on the training set. All random seeds were fixed at 42 for reproducibility.

**Model**	**Family**	**Key Hyperparameters**	**Scaling**
Logistic Regression	Linear	C = 1.0, penalty = L2, solver = SAGA, max_iter = 1000	Yes (StandardScaler)
KNN	Distance-based	k = 15, weights = uniform, metric = Minkowski	Yes (StandardScaler)
SVM (Linear)	Linear (kernel)	Linear kernel, C = 1.0, max_iter = 5000, Platt calibration (3-fold CV)	Yes (StandardScaler)
Decision Tree	Tree-based	max_depth = 10, criterion = Gini, splitter = best	No
Random Forest	Ensemble (bagging)	n_estimators = 200, max_depth = 15, criterion = Gini	No
XGBoost	Ensemble (boosting)	n_estimators = 300, max_depth = 6, learning_rate = 0.1, eval_metric = logloss	No
CatBoost	Ensemble (boosting)	iterations = 500, depth = 6, learning_rate = 0.1, eval_metric = AUC, ordered boosting	No
AdaBoost	Ensemble (boosting)	n_estimators = 200, learning_rate = 1.0, base estimator = Decision Tree (depth 1)	No
Gaussian Naïve Bayes	Probabilistic	var_smoothing = 1e−9 (default)	Yes (StandardScaler)
MLP	Neural network	hidden_layers = (128, 64), activation = ReLU, solver = Adam, early_stopping = True, validation_fraction = 0.1, max_iter = 300	Yes (StandardScaler)

Table S5. Complete feature definitions for all predictor variables. The first 21 features (Age through Transplant History) plus Complaint Category constitute the 23-variable baseline feature set. The provider feature was appended as either a target-encoded continuous variable (for scikit-learn models and XGBoost) or a native categorical (for CatBoost). EHR = electronic health record; EMS = emergency medical services.

Feature	Type	Description	Source
Age	Continuous	Patient age in years at time of ED visit	Demographics
Sex	Binary	1 = Female, 0 = Male	Demographics
ESI Level	Ordinal (1–5)	Emergency Severity Index triage acuity; 1 = most acute, 5 = least acute	Triage nurse
Complaint Category	Categorical	Chief complaint domain (e.g., Cardiac/Chest Pain, Respiratory, Abdominal/GI)	Triage nurse
SBP	Continuous	Systolic blood pressure (mmHg)	Triage vitals
DBP	Continuous	Diastolic blood pressure (mmHg)	Triage vitals
Heart Rate	Continuous	Heart rate (beats per minute)	Triage vitals
Respiratory Rate	Continuous	Respiratory rate (breaths per minute)	Triage vitals
SpO_2	Continuous	Peripheral oxygen saturation (%)	Triage vitals
Temperature	Continuous	Body temperature (°F)	Triage vitals
Ambulance Arrival	Binary	1 = arrived by ambulance/EMS, 0 = other	Registration
Wheelchair Arrival	Binary	1 = arrived by wheelchair, 0 = other	Registration
Heart Failure	Binary	History of heart failure on EHR problem list	Problem list
MI / CAD	Binary	History of myocardial infarction or coronary artery disease	Problem list
Diabetes	Binary	History of diabetes mellitus	Problem list
Chronic Kidney Disease	Binary	History of chronic kidney disease	Problem list
COPD / Asthma	Binary	History of chronic	Problem list

		obstructive pulmonary disease or asthma	
Cancer / Malignancy	Binary	Active cancer or malignancy	Problem list
TIA / Stroke	Binary	History of transient ischemic attack or cerebrovascular accident	Problem list
Pulmonary Embolism	Binary	History of pulmonary embolism	Problem list
Transplant History	Binary	History of solid organ transplant	Problem list
Provider (target-encoded)	Continuous	Training-set mean admission rate of the assigned provider (5-fold CV on training)	EHR
Provider (native categorical)	Categorical	Raw provider identity as native CatBoost categorical feature	EHR

Table S6. Overall cohort characteristics (not stratified by disposition) for training and test sets. The training cohort spans January 2020–December 2023; the test cohort spans January–December 2024. Admission rates were stable across time periods. IQR = interquartile range.

Characteristic	Training (n = 186,094)	Test (n = 58,151)
Total encounters	186,094	58,151
Admitted, n (%)	58,155 (31.3%)	18,443 (31.7%)
Age, median (IQR)	61 (42–74)	62 (42–75)
Female, n (%)	99,205 (53.3%)	31,485 (54.1%)
Ambulance arrival, n (%)	15,480 (8.3%)	4,790 (8.2%)
ESI 1, n (%)	1,288 (0.7%)	273 (0.5%)
ESI 2, n (%)	49,249 (26.5%)	13,982 (24.0%)
ESI 3, n (%)	111,205 (59.8%)	35,109 (60.4%)
ESI 4, n (%)	23,228 (12.5%)	8,501 (14.6%)
ESI 5, n (%)	1,066 (0.6%)	270 (0.5%)
SBP, mmHg, median (IQR)	128 (116–142)	129 (116–143)
DBP, mmHg, median (IQR)	77 (69–86)	77 (69–86)
Heart rate, bpm, median (IQR)	76 (66–86)	75 (66–85)
Respiratory rate, median (IQR)	16 (16–18)	16 (16–18)
SpO_2, %, median (IQR)	97 (96–99)	97 (95–98)
Temperature, °F, median (IQR)	98.1 (97.7–98.4)	98.0 (97.7–98.4)
Heart failure, n (%)	17,774 (9.6%)	4,884 (8.4%)
MI/CAD, n (%)	23,190 (12.5%)	6,919 (11.9%)
Diabetes, n (%)	38,400 (20.6%)	11,849 (20.4%)
CKD, n (%)	20,474 (11.0%)	5,716 (9.8%)
COPD/Asthma, n (%)	27,956 (15.0%)	8,893 (15.3%)
Cancer/Malignancy, n (%)	56,774 (30.5%)	17,972 (30.9%)
TIA/Stroke, n (%)	18,792 (10.1%)	5,399 (9.3%)
PE, n (%)	10,580 (5.7%)	3,525 (6.1%)
Transplant history, n (%)	7,561 (4.1%)	1,988 (3.4%)
Unique providers	66	49
